# Effects of overtreatment with different attachment positions on maxillary anchorage enhancement with clear aligners: a finite element analysis study

**DOI:** 10.1186/s12903-023-03340-0

**Published:** 2023-09-25

**Authors:** Shiyu Wang, Yangyang Huang, Dian Fan, Hao Liu, Changyong Yuan, Li Yang, Penglai Wang

**Affiliations:** 1 School of stomatology, Xuzhou Medical University, 221000 Xuzhou, China; 2 Department of Orthodontics, Affiliated Stomatological Hospital of Xuzhou Medical University, 221000 Xuzhou, China; 3 Department of Implantology, Affiliated Stomatological Hospital of Xuzhou Medical University, 221000 Xuzhou, China

**Keywords:** Clear aligners, Extraction cases, Overtreatment, Attachment, Finite element analysis

## Abstract

**Background:**

The effect of attachment positions on anchorage has not been fully explored. The aim of the present study is to analyze the effect of overtreatment with different anchorage positions on maxillary anchorage enhancement with clear aligners in extraction cases.

**Methods:**

Models of the maxilla and maxillary dentition were constructed and imported into SOLIDWORKS software to create periodontal ligament (PDL), clear aligners, and attachments. Attachment positions on second premolars included: without attachment (WOA), buccal attachment (BA), and bucco-palatal attachment (BPA). Overtreatment degrees were divided into five groups (0°, 1°, 2°, 3°, 4°) and added on the second premolars. The calculation and analysis of the displacement trends and stress were performed using ANSYS software.

**Results:**

Distal tipping and extrusion of the canines, and mesial tipping and intrusion of the posterior teeth occurred during retraction. A strong anchorage was achieved in cases of overtreatment of 2.8° with BA and 2.4° with BPA. Moreover, the BPA showed the best in achieving bodily control of the second premolars. When the overtreatment was performed, the canines and first molars also showed reduced tipping trends with second premolars attachments. And the stress on the PDL and the alveolar bone was significantly relieved and more evenly distributed in the BPA group.

**Conclusions:**

Overtreatment is an effective means for anchorage enhancement. However, the biomechanical effect of overtreatment differs across attachment positions. The BPA design performs at its best for stronger overtreatment effects with fewer adverse effects.

**Supplementary Information:**

The online version contains supplementary material available at 10.1186/s12903-023-03340-0.

## Background

The increasing demand of esthetic and comfortable appliances has prompted the development of clear aligners [1]. Compared to fixed appliances, clear aligners have advantages of better periodontal maintenance and lesser root resorption [2, 3]. Nowadays, clear aligners can effectively achieve various tooth movements, such as molar distalization and arch expansion [4, 5]. However, due to limitations in material properties, the control deficiency of clear aligners in complex tooth movements limits its application in extraction cases. A successful treatment depends on the well-controlled anchorage. However, anchorage loss is frequently seen and manifests as mesial tilt and intrusion of the posterior teeth [6, 7].

Overtreatment has been widely employed in clear aligners to improve its efficiency [8]. In extraction cases of the posterior region, overtreatment manifests as preset distal tipping [9]. During overtreatment, the attachment design is critical and is typically located on the buccal surfaces of the teeth. We previously found that the combined use of buccal and palatal attachments produced better results in molar intrusion than using single buccal attachment (BA) [10]. However, Ahmed et al. reported the opposite results in torque control [11]. Whether or not the bucco-palatal attachment (BPA) design is effective in anchorage control or further amplifies the effect of overtreatment remains unexplored.

Attachments are not curative in clinical practice. Anchorage loss occurs because of shortage of overtreatment. Dai et al. reported mesial tipping of the molars with overtreatment [12]. Due to the lack of relevant studies, the adequate amount of overtreatment has not been determined.

As an effective and reliable method, the finite element analysis (FEA) is widely used in biomechanical studies [13–15]. To simplify the analysis, a complicated assembly is divided into a finite number of units. The overall mechanical properties are obtained by analyzing and integrating the properties of each unit. Under a virtual clinical condition, the displacement tendencies of teeth and stress distributions of periodontal ligaments (PDLs) can be calculated and visualized [16], which helps to better understand the mechanisms of clear aligners.

The aim of the present study was to compare the biomechanical effect of overtreatment between three groups: without attachment (WOA), BA and BPA groups. We established a model of the maxillary dentition with first premolars extracted. Through the parameter settings and calculations, the relevant results were presented in the software. And the conclusions could be obtained in the subsequent data collection and analysis.

## Methods

### Sample selection and data acquisition

A 26-year-old man was recruited for the present study. Imaging data were obtained from the cone-beam computed tomography (CBCT) database. The study protocol was approved by the Ethical Committee of the Affiliated Stomatological Hospital of Xuzhou Medical University (2022-KY-004-01).

### Model establishment

**Fig. 1:**
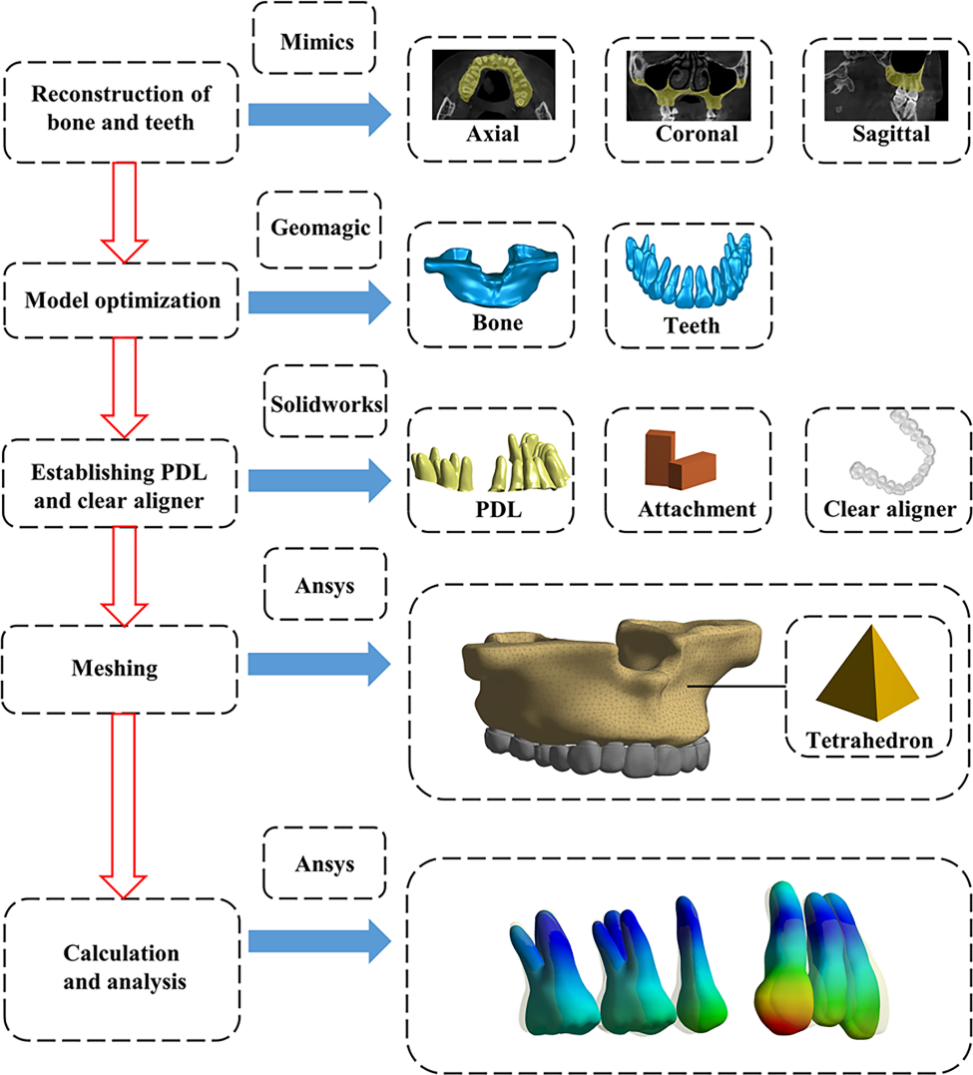
Flow chart of analysis

A total of 340 images were obtained from the archive and imported into MIMICS Research 21.0 (Materialize, Leuven, Belgium). Three-dimensional reconstruction of the maxilla and maxillary teeth was carried out through threshold separation and editing. Subsequently, the three-dimensional model was imported into GEOMAGIC Wrap 2017 (Geomagic, North Carolina, USA) for further optimization, including hole filling, spike removing and surface smoothening. The roots of the maxillary teeth were extended integrally and outwardly by 0.25 mm [17] to build the primary models of periodontal ligament (PDL). Using the Boolean operation, the final models of PDL were generated. Subsequently, each component was saved as Standard Tessellation Language files and imported into SOLIDWORKS 2019 (Dassault Systemes, Massachusetts, USA) to create clear aligners and attachments. The production of clear aligners was similar to that of the PDL. Clear aligners with a thickness of 0.75 mm were created through external extension of the crowns. Vertical rectangular attachments were added on the surfaces of the second premolars, first molars, and second molars, with a size of 3 mm×2 mm×1 mm [18]. To simulate the clinical setting, the first premolars were extracted from the model. Finally, components, including the maxilla, teeth, PDL, attachments and clear aligner, were assembled and imported into ANSYS Workbench 17.0 (ANSYS, Ltd, USA) for further analyses (Fig. 1).

### Material properties and model meshing

Model components were made of linear elastic, isotropic, and homogeneous materials. Table 1 shows the properties, including the elastic modulus and Poisson’s ratio, which were set in accordance with previous studies [19–22].

**Table 1:** Material properties

	Young’s modulus (MPa)	Poisson’s ratio
Teeth	19.6 × 10^3^	0.3
PDL	0.67	0.45
Bone	13.7 × 10^3^	0.3
Clear aligner	816	0.36
Attachment	12.5 × 10^3^	0.36

Based on the pre-defined mesh sizes, which were 2.0 mm for the maxillary bone, 0.8 mm for the maxillary dentition, 0.6 mm for the attachment, 0.8 mm for the PDL, and 0.6 mm for the clear aligner, the models were divided into tetrahedrons with finite size and number. Table 2 shows the number of nodes and elements.

**Table 2:** Number of nodes and elements of each component

		Nodes	Elements
Teeth		236,362	154,703
PDL		107,964	53,595
Bone		105,582	68,077
Clear aligner		205,730	118,875
Attachment	WOA	1549	665
	BA	2233	942
	BPA	3215	1386

### Boundary restrictions and contact conditions

To ensure that the maxilla remains stable under the loading condition, fixed support was placed on the superior region of bone with zero degrees of freedom in all directions. Bonded contacts were set between the bone and the PDL, PDL and the teeth, and teeth and the attachments to ensure that no displacement occurred under external forces. The contact conditions between clear aligners and teeth and attachments were frictional, with a Coulomb friction coefficient of 0.2 [23].

**Fig. 2:**
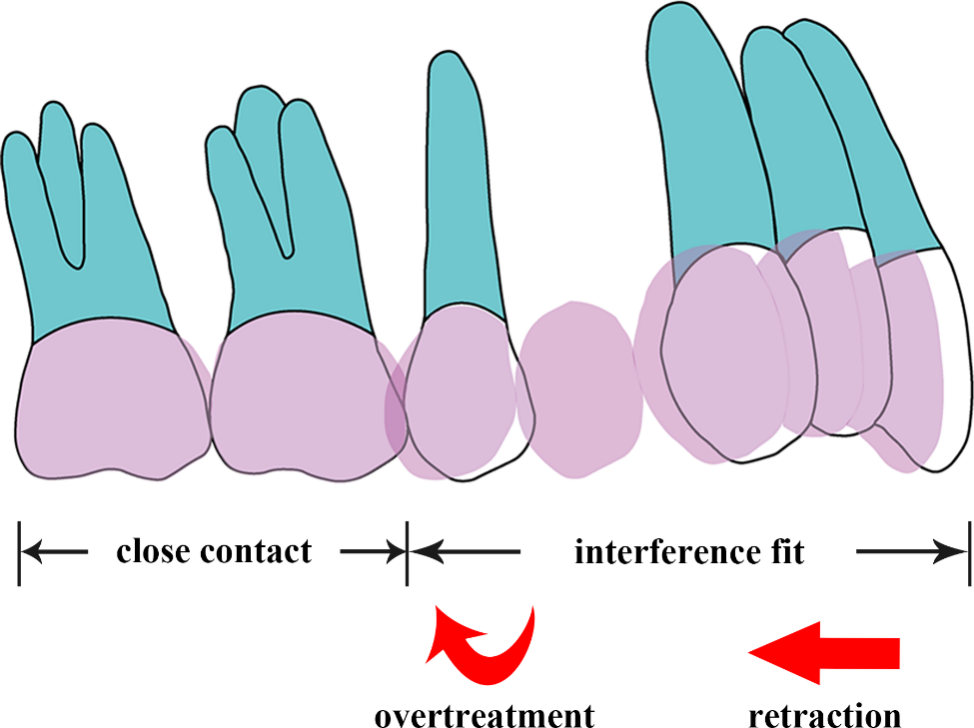
Loading method

The molars remained stationary in position and were in close contact with the aligners. However, considering that the amounts of retraction and overtreatment had been pre-programmed on the aligner, the anterior teeth and second premolar parts of the aligners were set with an interference fit (Fig. 2).

### Model design and groups

An en masse retraction of 0.25 mm was designed on the anterior aligner. Overtreatment was generated with a clockwise rotation of the aligner around the long axis of the second premolar, manifesting as an angle between the second premolar and the aligner in the sagittal view.

**Fig. 3:**
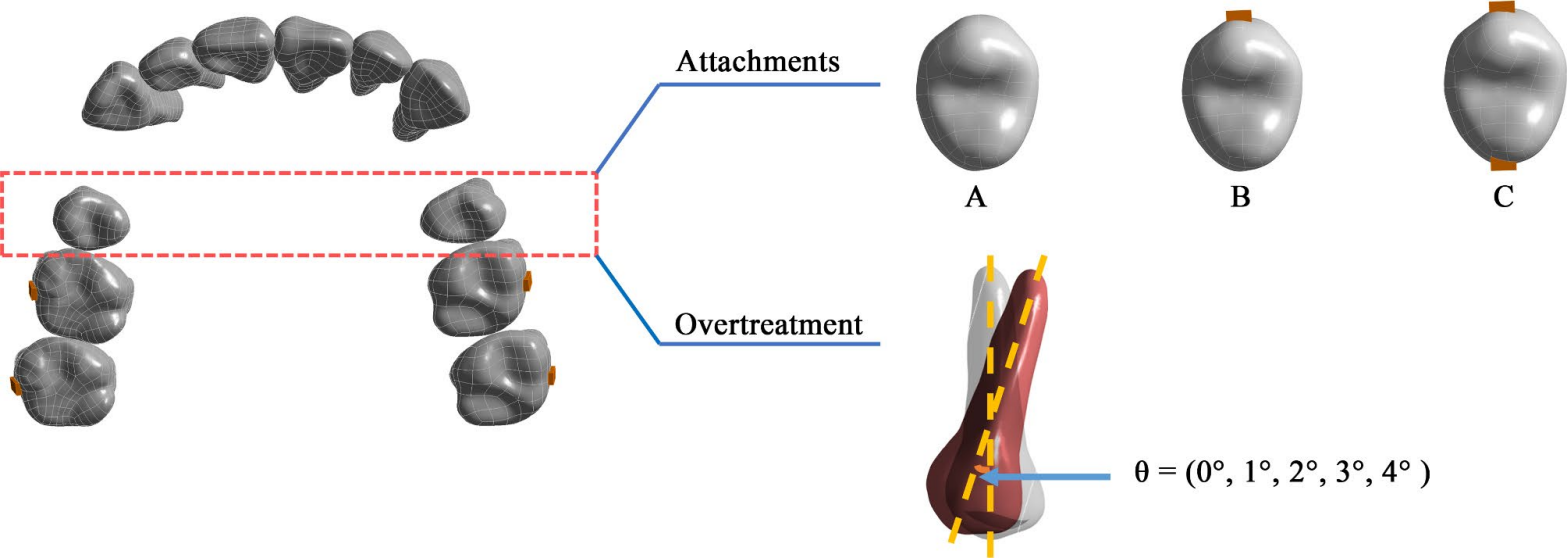
Fifteen groups. (**A**) Without attachment; (**B**) Buccal attachment; (**C**) Bucco-palatal attachment

According to the attachment positions of the second premolar, three groups were established: WOA, BA, and BPA (Fig. 3). Based on the degrees of overtreatment, each group was divided into five subgroups: 0°,1°,2°,3°, and 4°. Finally, a total of 15 (3 × 5 = 15) models were established.

### Coordinate system and measurement points

A coordinate system was built to measure the tooth movements. X-, Y-, and Z-axes represented the coronal, sagittal, and vertical directions, respectively. The positive direction pointed inward on the X-axis, forward on the Y-axis, and upward on the Z-axis.

The measurement points included: (1) cusps of the canines; (2) buccal and palatal cusps of the second premolars; (3) occlusal centers of the second premolars and molars.

## Results

### Differences in the displacement tendencies of the teeth with and without overtreatment

**Fig. 4:**
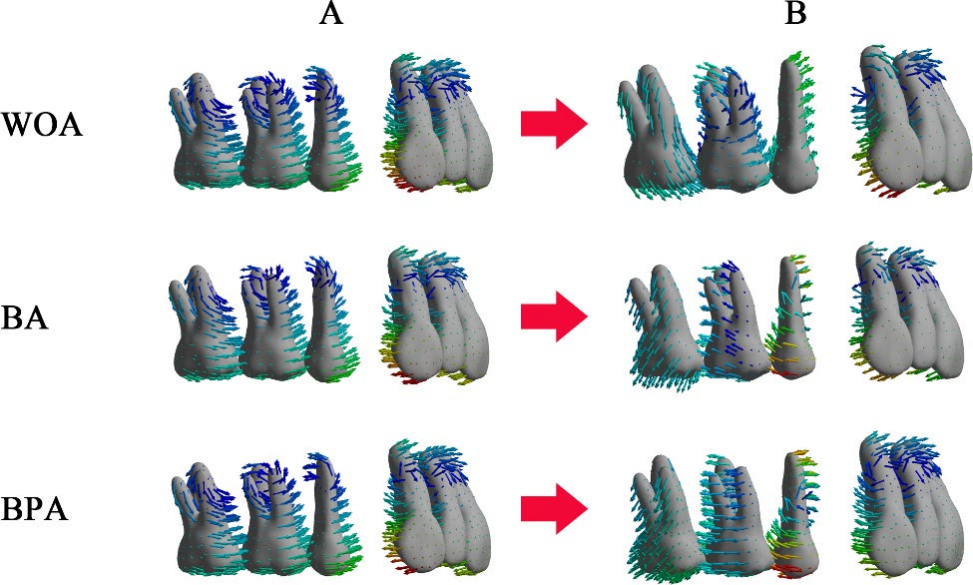
The displacement tendencies of the maxillary teeth. (**A**) Without overtreatment; (**B**) With overtreatment of 4°

Without overtreatment, the phenomena of lingual tipping and extrusion of the incisors, distal tipping and extrusion of the canines, mesial tipping and intrusion of the second premolars and first molars, and mesial tipping and extrusion of the second molars was observed. The displacement did not differ significantly among the three groups. With the overtreatment of 4°, the anterior teeth showed the similar movements. However, the tipping directions of the posterior teeth shifted from mesial to distal in the BA and BPA group, while that of the molars remained mesial in the WOA group (Fig. 4).

### Differences in the displacement tendencies of the second premolars

**Fig. 5:**
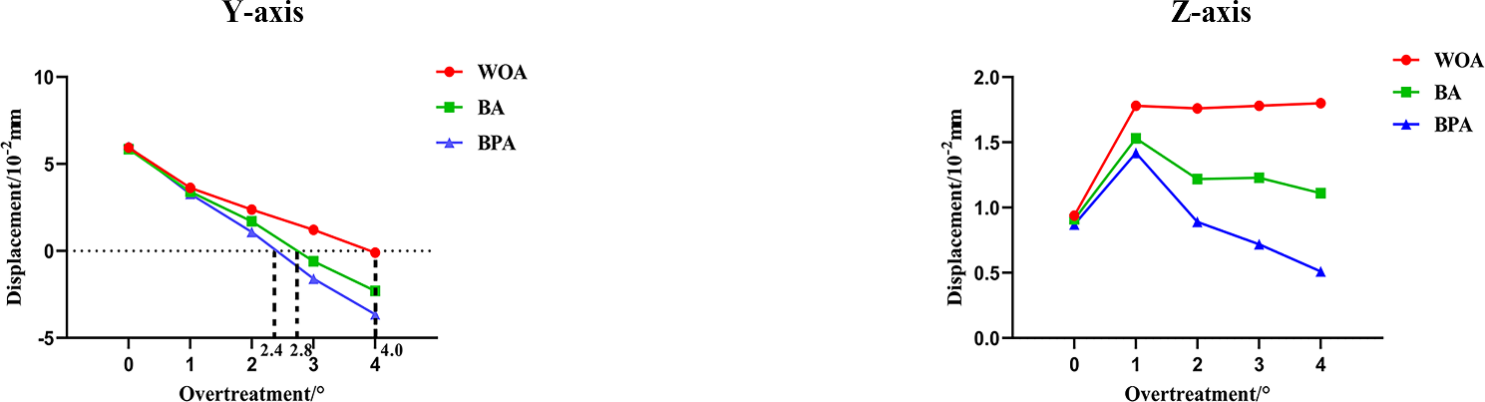
The sagittal and vertical displacements of the second premolars as the overtreatment was increased

The sagittal displacement steadily decreased as the overtreatment was increased. And the BPA group showed the highest rate. The displacement reached zero when the overtreatment was 4° with WOA, 2.8° with BA, and 2.4° with BPA (Fig. 5).

With no overtreatment, the WOA group displayed the most movement, while the BPA group displayed the least (Fig. 5). A rapid increase occurred when the overtreatment was 1°. Then the value maintained constant in the WOA group, but decreased in the BA and BPA groups. In the BPA group, the amount of intrusion was almost twice as smaller than that in the BA group.

### Differences in the buccal and palatal movements of the second premolars

**Fig. 6:**
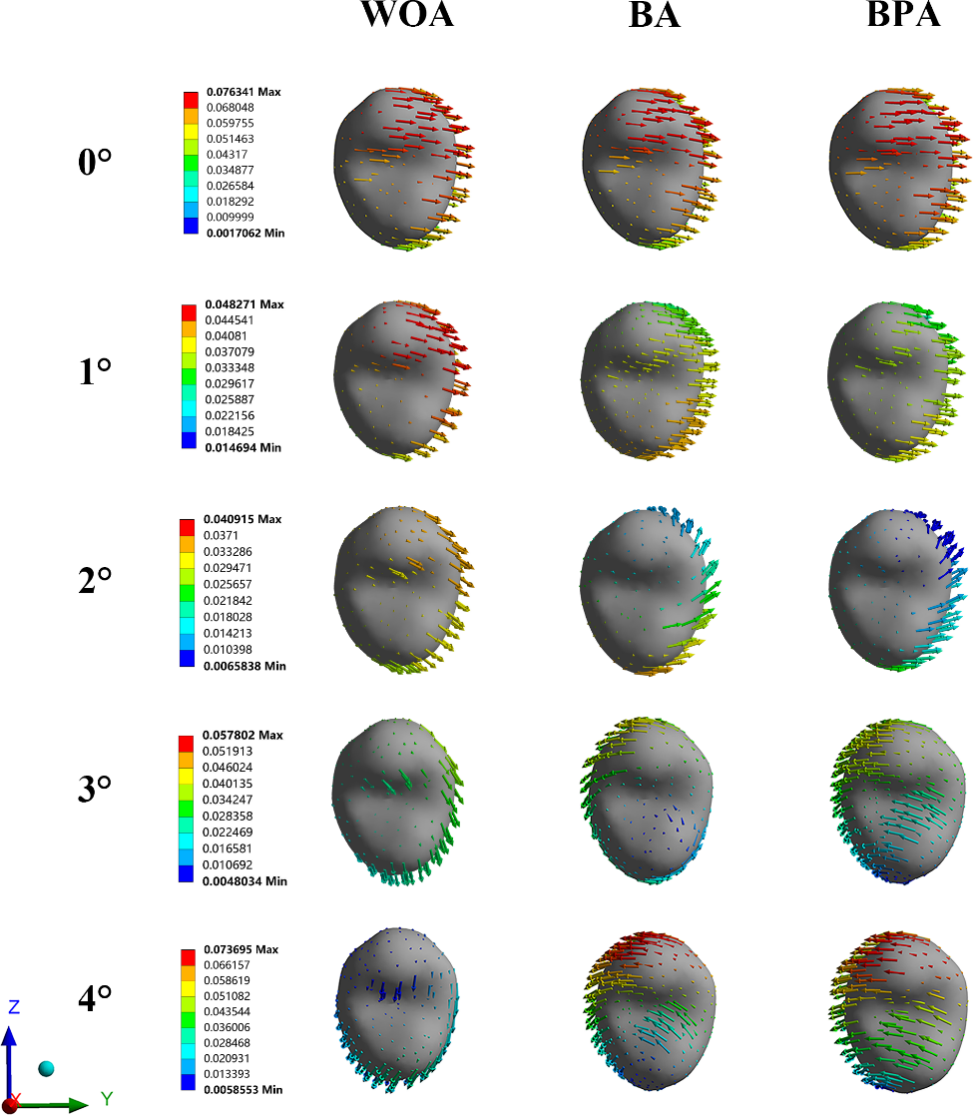
The displacement tendencies of the second premolars in the vertical view

**Fig. 7:**
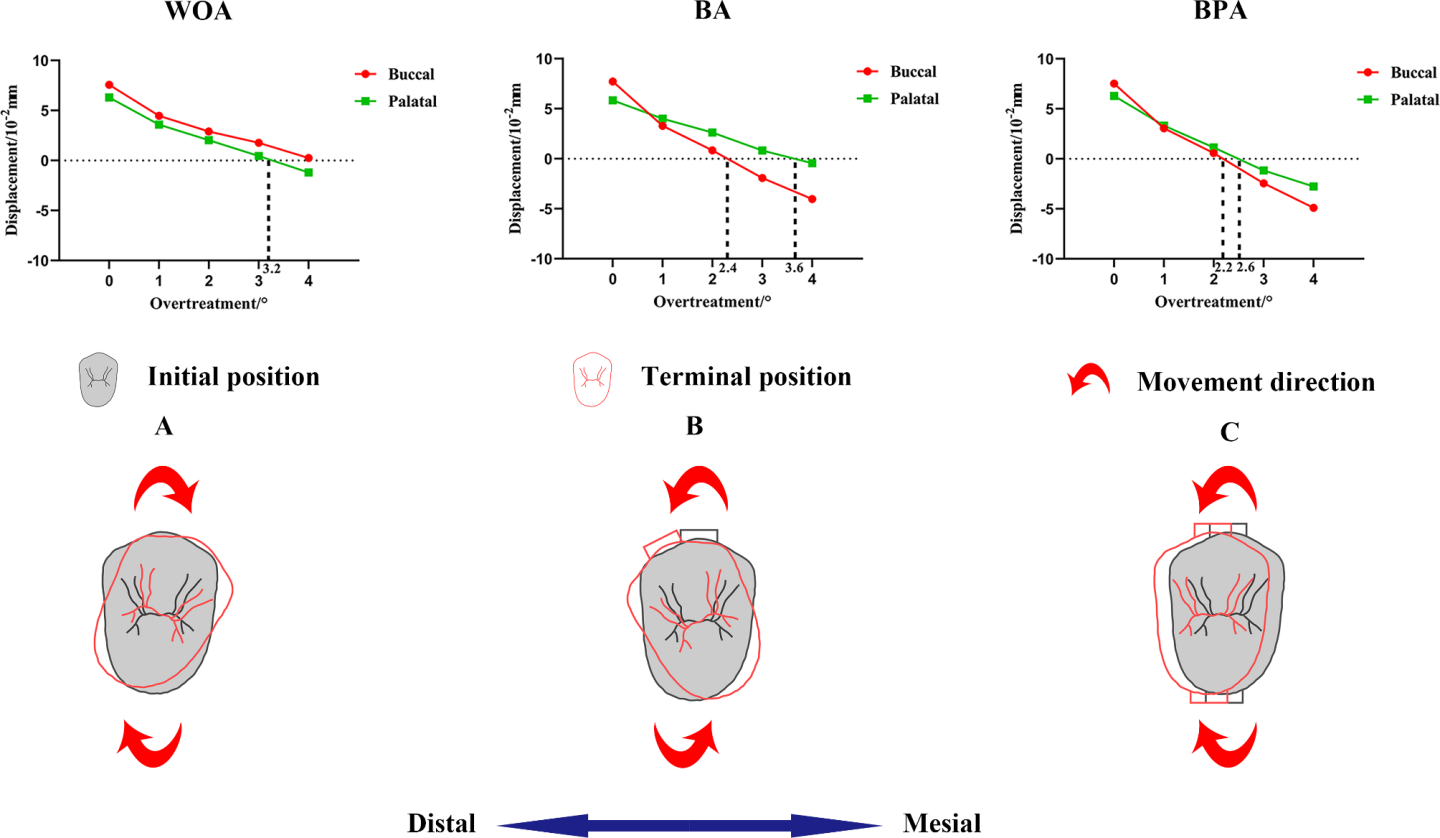
Movement directions of the buccal and palatal cusps of the second premolars. (**A**) WOA with 4° overtreatment; (**B**) BA with 3° overtreatment; (**C**) BPA with 3° overtreatment

The changes of buccal and palatal displacements were not the same (Fig. 6). The buccal cusp stopped mesial tipping when the overtreatment was 2.2° with BA and 2.4° with BPA. The palatal displacement reached zero until the overtreatment was 3.2° with WOA, 3.6° with BA, and 2.6° with BPA (Fig. 7). The difference between the two cusps in the BA group exceed 1°, while that in the BPA group was just 0.4°.

The buccal and palatal cusps even moved in opposite directions under certain degrees of overtreatment (Fig. 7). For WOA group, with 4° overtreatment, the buccal cusp moved forward, while the palatal cusp moved backward. For BA group, with 3°overtreatment, the buccal cusp moved backward, while the palatal cusp moved forward. However, in the BPA group, the buccal and palatal cusps consistently moved in the same direction.

### Changes in the displacement tendencies of the adjacent teeth

**Fig. 8:**
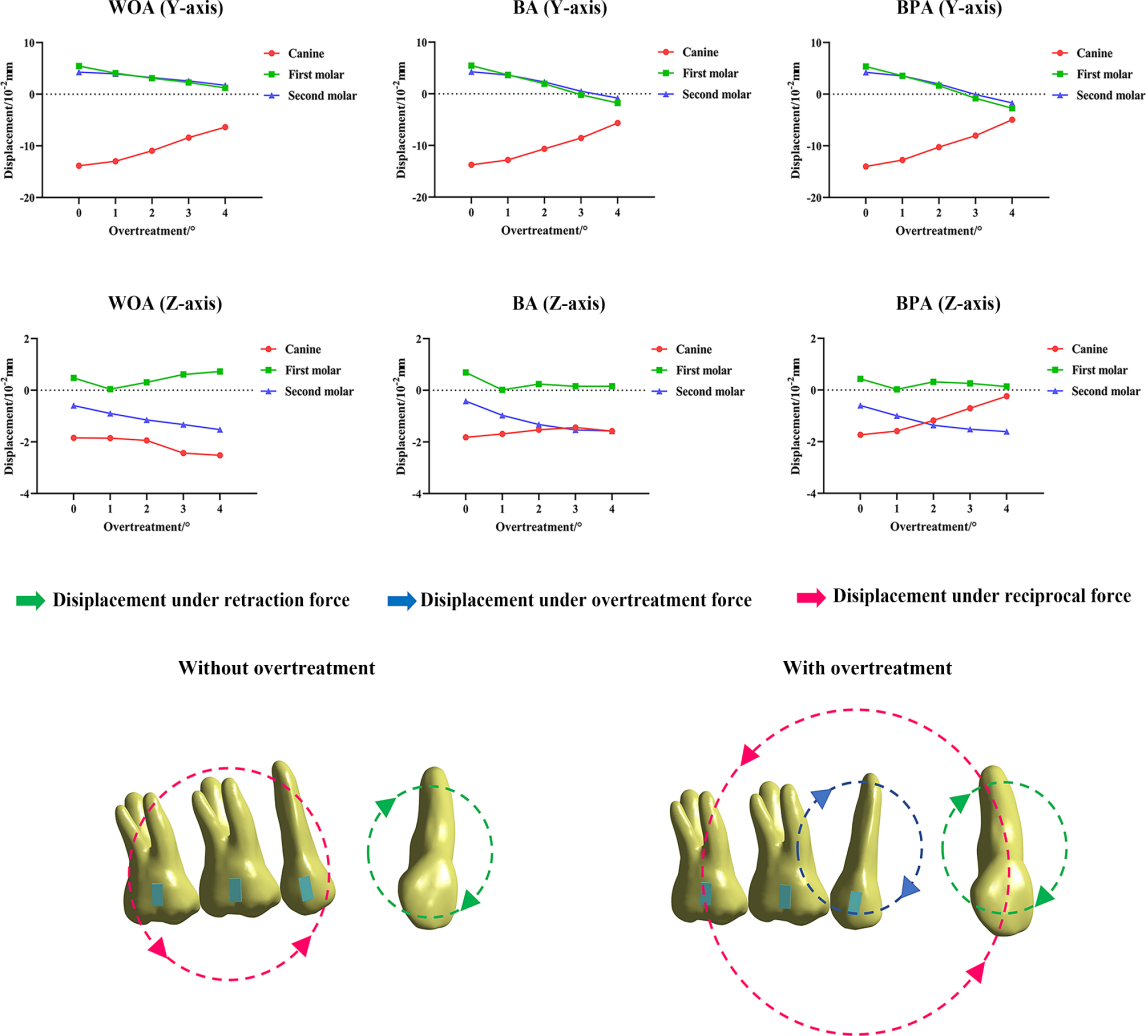
Changes of displacement tendencies of the canines, first molars and second molars

With the overtreatment on the second premolars, the movements of the canines and molars were also changed (Fig. 8). The sagittal displacements were reduced. And the canines and first molars showed lesser displacements with the second premolar attachments, which was more significant with BPA. However, the WOA group showed more displacements. Unlike the canines and first molars, the second molars exhibited extrusion all the time.

Figure 8 shows the performance of the teeth in the BPA group. Without the overtreatment, the canines rotated under the retraction force, while the reciprocal force moved the posterior teeth forward and upward. When the overtreatment was added in a clockwise direction, extrusion and distal tipping of the canines, and intrusion and mesial tipping of the molars decreased under the reciprocal force in an anticlockwise direction.

### Differences in the stress distributions in the PDL of the second premolars

**Fig. 9:**
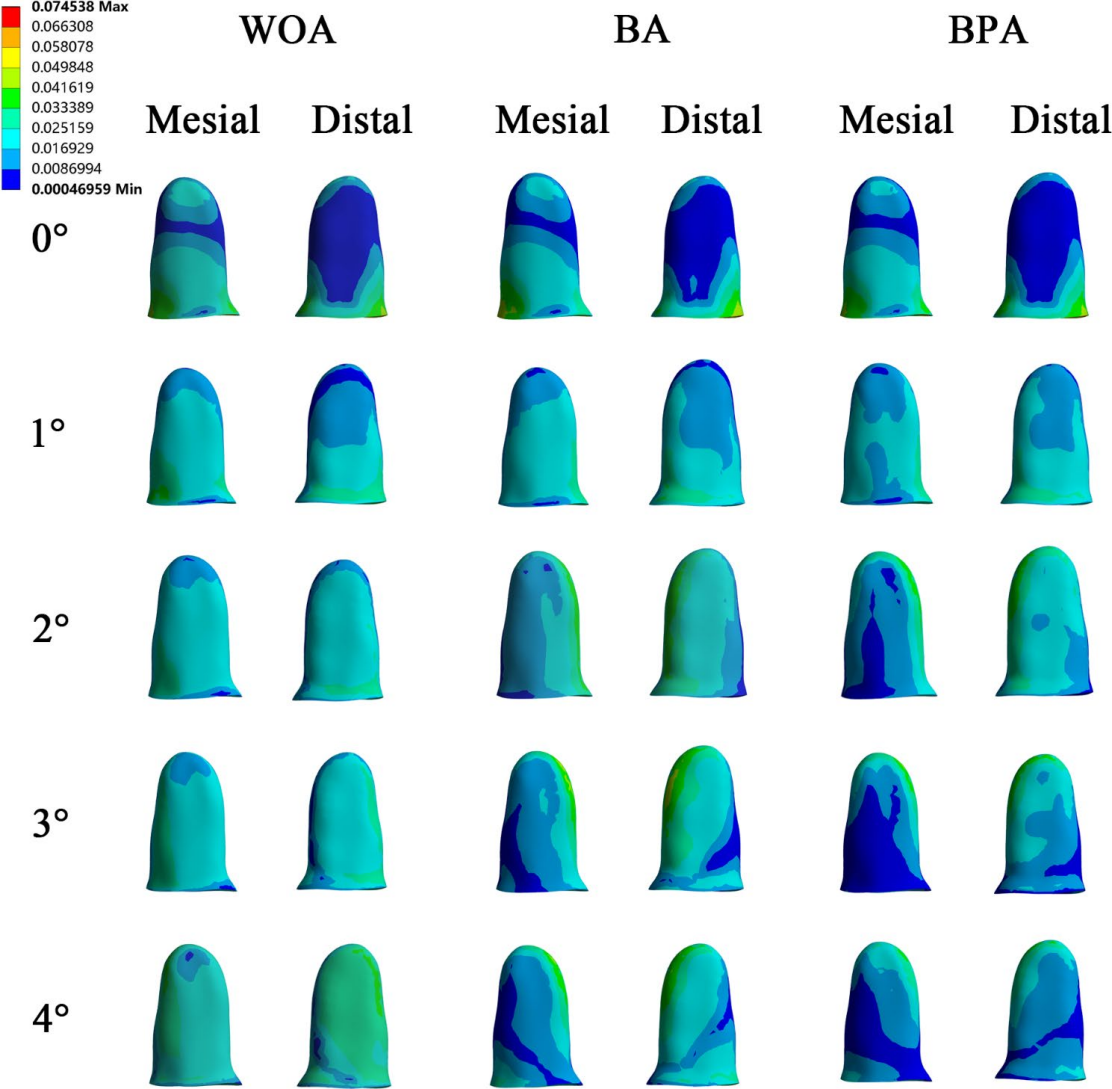
Von mises stress on the PDL of the second premolars

Without overtreatment, the maximum stress of the PDL was observed in the cervical and apical regions. When the overtreatment was added, the stress was widely distributed in the WOA group, while the stress was more concentrated on the distal surfaces and apical regions in the BA group. However, in the BPA group, the stress was even-distributed and significantly relieved (Fig. 9).

**Fig. 10:**
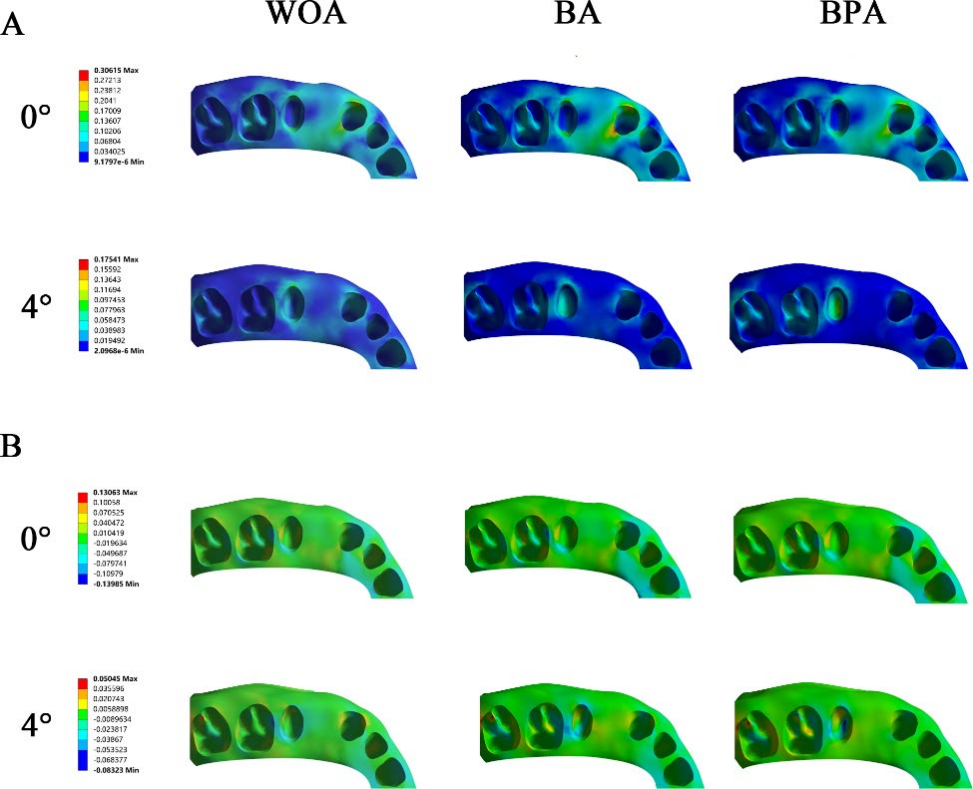
The stress distribution on the alveolar bone. (**A**) Von mises stress of the bone; (**B**) Compressive and tensile stresses of the bone

Without overtreatment, the stress was concentrated on the buccal and palatal alveolar ridges. And the distal ridges were under tensile stress. With the overtreatment, the stress in the BA and BPA group was mainly distributed in the apical regions. The mesial ridge and disto-apical regions were under tensile stress. The distal ridge and mesio-apical regions were under compressive stress (Fig. 10). However, compared to the BA group, the BPA group exhibited more even stress distribution.

## Discussion

FEA, an effective and reliable tool in biomechanics, was employed in the present study for a visual analysis. This study explored how attachment positions affected anchorage in extraction cases. Moreover, we ascertained the adequate amount of overtreatment for maxillary teeth. If the amount of retraction was 0.25 mm, the appropriate degrees of overtreatment on maxillary second premolars were 2.8° with BA and 2.4° with BPA.

Interference fit was applied to simulate the force loading [22, 24, 25]. During treatment planning, the amount of tooth movements was pre-designed in the aligners. In other words, the surfaces of the teeth and aligners were not in close contact with each other. Once the aligner was placed, the interference between them generated a force perpendicular to the contact surfaces. Further, the teeth moved toward the target positions due to the deformation of the aligners.

In the present study, we selected the second premolars as the subject. This design was based on certain considerations. First, the second premolars were adjacent to the extraction sites, presenting the highest anchorage loss [22, 26]. Second, the anchorage of the distal molars could be protected with the overtreatment added on the second premolars. In addition, designing BPA on each posterior tooth did not conform with clinical practice. Although increasing attachments would improve the efficacy [27], insertion and removal of the aligner might become difficult.

In extraction cases, the methods for anchorage enhancement include two-step retraction [28], overtreatment [9], intermaxillary traction [29], and mini-screw [26]. Compared to the potential joint injury with intermaxillary traction [30] and invasive mini-screw implantation, the noninvasive overtreatment can be directly designed into the treatment protocol and achieved by replacing aligners step-wise. In clear aligners, the anchorage overtreatment manifests as an angle between the inner surface of the aligner and the outer surface of the teeth. However, this means that the aligner is not in close contact with the teeth, which reduces retention and effectiveness. As a frequently used tool to enhance retention force [31], the application of attachments can improve this situation. The regular shape of the attachment helps exert orthodontic forces and facilitates the realization of complicated movements, such as bodily movement [32]. Therefore, attachments play a crucial role in designing anchorage overtreatment.

The buccal and palatal surfaces of teeth were concurrently covered by the aligners. However, the bodily movements were not achieved. This might be related to the tooth morphology. The contact area of the second premolar was close to the buccal surface, leading to the buccal aligner having a less coverage area and retention. Further, the flat buccal surface was conducive to force transmission. Therefore, if no attachment was designed, the anchorage of the palatal side was stronger than that of the buccal side. However, when BA was introduced, the control force of aligner to the buccal surface increased significantly. But this, in turn, created a new imbalance. The distinct difference between the buccal and palatal displacements showed the necessity of the palatal attachment. With the combined use of buccal and palatal attachments, the mesial displacement of the palatal cusp also decreased significantly, and the differences between the two cusps were the least among the three groups. Thus, BPA showed the best performance in bodily control of the second premolars.

With no overtreatment, the second premolars displacement in the BA group was similar to that in the BPA group. However, the BPA group showed the least displacement once the overtreatment was added. Significant differences existed between them. The BPA design enhanced the control force of the aligner to the teeth. And compared to the smooth surfaces, the sharp edges of attachments also increased the efficiency [27]. Therefore, BPA performed the best in anchorage enhancement.

The second premolars were in contact with the first molars. When the second premolars began to tip distally, the first molars received the distal force and moved distally as a result. However, as the force gradually attenuated due to friction [33], the magnitude of distal displacement of the first molars was less than that of the second premolars. Moreover, when the overtreatment in the disto-occlusal direction was added, a reciprocal force in the mesio-apical direction was also inevitably generated on teeth and transmitted to the aligner. In the canine region, the force could counter the distal tipping and intrusion tendencies of the canines. However, if no attachment was designed, the amount of overtreatment was not sufficient. As the second premolars remained tipping mesially, the tipping tendencies of the canines and first molars could not be reduced. This result suggested that the overtreatment designed on the second premolar with attachments might also be useful in preventing the roller coaster effect.

Although several studies have revealed that excessive force increases the risk of root resorption and alveolar defect [22, 34–36], the present study is a FEA study and the inference should be prudent. Therefore, we just discussed the stress distribution. The differences in stress distribution might be attributed to the different movement patterns of the teeth. Without overtreatment, the mesial tipping of the second premolars caused the stress concentration on the cervical and apical areas [37]. With overtreatment, the crown and root moved mesially together in the WOA group. For BA, the buccal crown moved distally while the root moved mesially. However, the PDL and alveolar bone showed reduced and even-distributed stress with BPA design, which could be attributed to the bodily movements of buccal and palatal crowns. The results also suggested that the BPA design have potential to achieve better stress distribution.

This study successfully utilized the interference fit to conduct the analysis. The obtained results indicated that the interference fit was a feasible and effective loading method. The optimal overtreatment degrees and BPA design could also provide a reference for treatment planning. However, the limitations of this study could not be ignored. In clinical practice, the four premolars extraction cases were more common, but we failed to construct the mandible. Liu et al. reported that less anchorage loss occurred in mandibular teeth [38]. We assumed that less overtreatment is needed for the mandibular anchorage. If the Class II elastics is applied, the distal force on the upper aligner could reduce the maxillary anchorage overtreatment required, while the mesial force on the mandibular first molar might increase the mandibular anchorage overtreatment. However, the lingual elastics design might have potential to reduce the adverse effect of Class II elastics [18]. In addition, the actual clinical settings cannot be replaced by virtual simulation. More work is needed to substantiate the conclusion. The practicality and effectiveness of this study should be examined in further clinical cases.

## Conclusions

1. The overtreatment was an effective method to reduce the anchorage loss. And the effect could be amplified by attachments.
2. Under the retraction of 0.25 mm, the strong anchorage was achieved at an overtreatment of 2.8° with BA and 2.4° with BPA.
3. The BPA design could achieve the bodily control of the buccal and palatal movements of the teeth.
4. The overtreatment with BPA on the second premolars could reduce the tipping tendencies of the canines and molars.

### Electronic supplementary material

Below is the link to the electronic supplementary material.

Supplementary Material 1

Supplementary Material 2

Supplementary Material 3

Supplementary Material 4

## Data Availability

The datasets used and analyzed during the current study are available from the corresponding author upon reasonable request.

## Abbreviations

BA: buccal attachment
BPA: bucco-palatal attachment
FEA: finite element analysis
PDL: periodontal ligament
WOA: without attachment
