# Supplementary material for: Effects of overtreatment with different attachment positions on maxillary anchorage enhancement with clear aligners: a finite element analysis study
Source: BMC Oral Health. 2023 Sep 25;23:693. doi: 10.1186/s12903-023-03340-0 (PMC10521390; doi:10.1186/s12903-023-03340-0)
Supplement: Supplementary file 1 — Supplementary Material 1 [file 12903_2023_3340_MOESM1_ESM.docx]

| WOA |  | 0° | | 1° | | 2° | | 3° | 4° |
| --- | --- | --- | --- | --- | --- | --- | --- | --- | --- |
| Central incisor | x-axis | -0.17 | -0.10 | | 0.15 | | 0.83 | | 1.23 |
|  | y-axis | -8.94 | -8.45 | | -7.27 | | -5.91 | | -4.59 |
|  | z-axis | -2.23 | -2.10 | | -1.82 | | -1.63 | | -1.43 |
| Lateral incisor | x-axis | 0.10 | 0.16 | | 0.39 | | 0.92 | | 1.08 |
|  | y-axis | -9.01 | -8.53 | | -7.35 | | -5.69 | | -4.05 |
|  | z-axis | -1.11 | -1.06 | | -0.98 | | -1.03 | | -0.89 |
| Canine | x-axis | 3.08 | 2.87 | | 2.48 | | 2.62 | | 2.30 |
|  | y-axis | -13.83 | -12.96 | | -10.95 | | -8.38 | | -6.36 |
|  | z-axis | -1.84 | -1.85 | | -1.94 | | -2.43 | | -2.52 |
| Second premolar | x-axis | -0.86 | -1.03 | | -1.28 | | -1.69 | | -1.80 |
|  | y-axis | 6.31 | 3.68 | | 2.32 | | 1.11 | | -0.30 |
|  | z-axis | 0.94 | 1.78 | | 1.76 | | 1.78 | | 1.80 |
| First molar | x-axis | -0.41 | -0.33 | | -0.47 | | -0.57 | | -0.53 |
|  | y-axis | 5.45 | 4.10 | | 3.12 | | 2.29 | | 1.24 |
|  | z-axis | 0.48 | 0.04 | | 0.31 | | 0.61 | | 0.73 |
| Second molar | x-axis | 0.20 | 0.01 | | -0.18 | | -0.42 | | -0.58 |
|  | y-axis | 4.30 | 3.95 | | 3.21 | | 2.58 | | 1.74 |
|  | z-axis | -0.59 | -0.90 | | -1.15 | | -1.33 | | -1.52 |

**Supplementary file 1.** Three-dimensional displacement values for the maxillary teeth in the WOA group (10^-2^mm).
