# Supplementary material for: Effects of overtreatment with different attachment positions on maxillary anchorage enhancement with clear aligners: a finite element analysis study
Source: BMC Oral Health. 2023 Sep 25;23:693. doi: 10.1186/s12903-023-03340-0 (PMC10521390; doi:10.1186/s12903-023-03340-0)
Supplement: Supplementary file 2 — Supplementary Material 2 [file 12903_2023_3340_MOESM2_ESM.docx]

| BA |  | 0° | | 1° | | 2° | | 3° | 4° |
| --- | --- | --- | --- | --- | --- | --- | --- | --- | --- |
| Central incisor | x-axis | -0.14 | -0.13 | | 0.09 | | 0.33 | | 0.79 |
|  | y-axis | -8.95 | -8.36 | | -7.06 | | -5.79 | | -3.80 |
|  | z-axis | -2.25 | -2.12 | | -1.85 | | -1.62 | | -1.31 |
| Lateral incisor | x-axis | 0.11 | 0.15 | | 0.36 | | 0.60 | | 0.98 |
|  | y-axis | -8.95 | -8.40 | | -7.11 | | -5.82 | | -3.89 |
|  | z-axis | -1.12 | -1.04 | | -0.94 | | -0.87 | | -0.76 |
| Canine | x-axis | 3.12 | 2.89 | | 2.53 | | 2.30 | | 2.29 |
|  | y-axis | -13.74 | -12.78 | | -10.64 | | -8.53 | | -5.63 |
|  | z-axis | -1.82 | -1.69 | | -1.53 | | -1.44 | | -1.58 |
| Second premolar | x-axis | -0.97 | -0.62 | | -0.01 | | 0.27 | | 0.91 |
|  | y-axis | 6.25 | 3.34 | | 1.39 | | -1.17 | | -3.05 |
|  | z-axis | 0.91 | 1.53 | | 1.22 | | 1.23 | | 1.11 |
| First molar | x-axis | -0.44 | -0.22 | | -0.19 | | 0.32 | | 0.58 |
|  | y-axis | 5.45 | 3.69 | | 1.96 | | -0.18 | | -1.76 |
|  | z-axis | 0.69 | 0.02 | | 0.24 | | 0.16 | | 0.16 |
| Second molar | x-axis | 0.17 | 0.11 | | 0.10 | | 0.07 | | -0.09 |
|  | y-axis | 4.30 | 3.64 | | 2.31 | | 0.52 | | -0.81 |
|  | z-axis | -0.42 | -0.97 | | -1.32 | | -1.53 | | -1.57 |

**Supplementary file 2.** Three-dimensional displacement values for the maxillary teeth in the BA group (10^-2^mm).
