# Supplementary material for: Effects of overtreatment with different attachment positions on maxillary anchorage enhancement with clear aligners: a finite element analysis study
Source: BMC Oral Health. 2023 Sep 25;23:693. doi: 10.1186/s12903-023-03340-0 (PMC10521390; doi:10.1186/s12903-023-03340-0)
Supplement: Supplementary file 3 — Supplementary Material 3 [file 12903_2023_3340_MOESM3_ESM.docx]

| BPA |  | 0° | | 1° | | 2° | | 3° | 4° |
| --- | --- | --- | --- | --- | --- | --- | --- | --- | --- |
| Central incisor | x-axis | -0.20 | -0.09 | | -0.04 | | -0.03 | | 0.05 |
|  | y-axis | -9.03 | -8.42 | | -7.16 | | -6.02 | | -4.15 |
|  | z-axis | -2.25 | -2.12 | | -1.80 | | -1.53 | | -1.13 |
| Lateral incisor | x-axis | 0.05 | 0.14 | | 0.14 | | 0.10 | | 0.09 |
|  | y-axis | -9.09 | -8.41 | | -7.01 | | -5.75 | | -3.71 |
|  | z-axis | -1.12 | -1.05 | | -0.86 | | -0.67 | | -0.42 |
| Canine | x-axis | 3.08 | 2.77 | | 2.00 | | 1.24 | | 0.64 |
|  | y-axis | -13.98 | -12.74 | | -10.24 | | -8.02 | | -4.94 |
|  | z-axis | -1.73 | -1.59 | | -1.18 | | -0.70 | | -0.24 |
| Second premolar | x-axis | -0.77 | -0.59 | | -0.18 | | 0.38 | | 1.11 |
|  | y-axis | 6.35 | 3.12 | | 0.82 | | -2.13 | | -4.37 |
|  | z-axis | 0.87 | 1.42 | | 0.89 | | 0.72 | | 0.51 |
| First molar | x-axis | -0.37 | -0.31 | | -0.44 | | -0.29 | | -0.18 |
|  | y-axis | 5.36 | 3.57 | | 1.65 | | -0.78 | | -2.71 |
|  | z-axis | 0.43 | 0.03 | | 0.32 | | 0.26 | | 0.14 |
| Second molar | x-axis | 0.11 | 0.00 | | -0.26 | | -0.62 | | -0.89 |
|  | y-axis | 4.23 | 3.53 | | 1.99 | | -0.07 | | -1.68 |
|  | z-axis | -0.60 | -1.00 | | -1.36 | | -1.52 | | -1.61 |

**Supplementary file 3.** Three-dimensional displacement values for the maxillary teeth in the BPA group (10^-2^mm).
