# Supplementary material for: Effects of overtreatment with different attachment positions on maxillary anchorage enhancement with clear aligners: a finite element analysis study
Source: BMC Oral Health. 2023 Sep 25;23:693. doi: 10.1186/s12903-023-03340-0 (PMC10521390; doi:10.1186/s12903-023-03340-0)
Supplement: Supplementary file 4 — Supplementary Material 4 [file 12903_2023_3340_MOESM4_ESM.docx]

|  |  | 0° | | 1° | | 2° | | 3° | 4° |
| --- | --- | --- | --- | --- | --- | --- | --- | --- | --- |
| WOA | buccal | 7.58 | 4.48 | | 2.91 | | 1.78 | | 0.26 |
|  | palatal | 6.31 | 3.59 | | 2.04 | | 0.45 | | -1.20 |
| BA | buccal | 7.72 | 3.29 | | 0.84 | | -1.92 | | -4.02 |
|  | palatal | 5.84 | 4.01 | | 2.63 | | 0.82 | | -0.44 |
| BPA | buccal | 7.53 | 3.05 | | 0.58 | | -2.45 | | -4.90 |
|  | palatal | 6.29 | 3.33 | | 1.14 | | -1.16 | | -2.76 |

**Supplementary file 4.** The buccal and palatal displacement values for the second premolars among the three groups (10^-2^mm).
